# Oesophageal tissue engineering: optimisation of stereotactic robotic cell injection in decellularised oesophageal scaffolds

**DOI:** 10.1007/s00383-026-06309-6

**Published:** 2026-02-20

**Authors:** Koji Yamada, Julia Perea Paizal, Elena Canovai, Casper Orens, Ilaria Marcoccio, Natalie Durkin, Lorenzo Caciolli, Satoshi Ieiri, Simon Eaton, Sara Mantero, Paolo De Coppi, Marco Pellegrini

**Affiliations:** 1 Stem Cell and Regenerative Medicine Section, Developmental Biology and Cancer Research and Teaching Department, Zayed Centre for Research into Rare Disease in Children, UCL Great Ormond Street Institute of Child Health, London, UK; 2 Department of Pediatric Surgery, Research Field in Medicine and Health Sciences, Medical and Dental Sciences Area, Research and Education Assembly, Kagoshima University, Kagoshima, Japan; 3 Department of Pediatric Surgery, Fondazione IRCCS Ca’ Granda, Ospedale Maggiore Policlinico, University of Milan, Milan, Italy; 4 Department of Chemistry, Materials and Chemical Engineering “Giulio Natta”, Politecnico di Milano, Milan, Italy; 5 NIHR GOSH Biomedical Research Centre, Great Ormond Street Hospital, London, UK; 6 Specialist Neonatal and Paediatric Surgery, Great Ormond Street Hospital for Children NHS Foundation Trust, London, UK

**Keywords:** Tissue engineering, Oesophageal atresia, Recellularisation, Robotic injection, Tissue clearing

## Abstract

**Purpose:**

Oesophageal substitution following atresia repair, caustic damage or cancer of the oesophagus can be challenging. We and others are working on engineering oesophageal tissue using a combination of decellularised oesophagi and cell injection. So far this has been achieved using highly operator-dependent techniques. This study aimed to establish a reproducible method for cell delivery into scaffolds.

**Methods:**

To improve consistency, a stereotaxic robotic platform was adapted to deliver a suspension of porcine gelatin and cells in a 1:1 ratio. The scaffold was mounted on a 3D-printed rod linked to a stepper motor, enabling automated 36° rotation for circumferential coverage. Two circumferential rows, each rotated 36°, with 3 − 2 points at 3-mm intervals, ensured even seeding. Injection depth was calibrated to target the inner layer.

**Results:**

Cells injected robotically remained viable, with no significant difference from manual injection. Post-injection analyses confirmed cell viability and distribution within the scaffold.

**Conclusion:**

Automated robotic injection provides a reliable, reproducible alternative to manual methods, reducing operator bias.

**Supplementary Information:**

The online version contains supplementary material available at 10.1007/s00383-026-06309-6.

## Introduction

Oesophageal atresia (OA) is a rare malformation, occurring in about 1 in 3,500–4,500 live births [1–3]. The condition is classified according to the presence and location of a tracheo-oesophageal fistula [4]. The initial operation is the primary anastomosis of the oesophageal ends with ligation of the fistula. However, this is not possible in long-gap cases, where the distance between the upper and lower oesophageal segments is too long [5]. In addition to OA, other conditions such as caustic strictures or oesophageal cancer can result in a requirement for bridging the two ends of the oesophagus.

Treatment for long-gap OA, including staged anastomosis or replacement with autologous intestinal or gastric segments, is associated with significant risk, including severe postoperative complications. Although none of them can be regarded as ideal, they remain the only available options at present. We have therefore focused on transplantation using a tissue-engineered (TE) oesophageal substitute. Our team has pioneered a personalised, size-matched TE approach using decellularised porcine oesophagi as scaffolds, repopulated with mesenchymal cells (i.e., mesoangioblasts (MABs), and fibroblasts (FBs)). These grafts are matured in custom-made bioreactors before transplantation [6, 7].

**Fig. 1:**
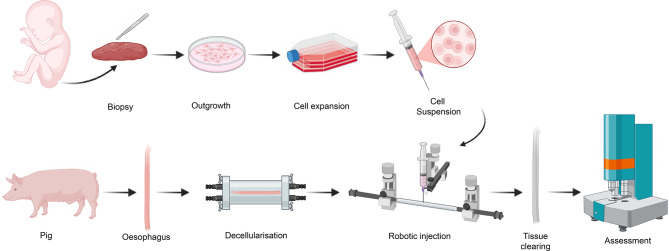
Experimental design. Skeletal muscle biopsy was collected from a Sistrunk procedure, cells were isolated, expanded and characterised prior to injection. Oesophagi were harvested from pigs, decellularised, and injected with cell suspension using a robotic system. After injection, oesophagi were subjected to tissue clearing, followed by assessment of cell viability and retention within the tissue

In our previous work, cell delivery into the decellularised scaffold was performed by manual microinjection. This technique is highly operator-dependent and lacks reproducibility, which might result in limited stable production. To address these limitations, we developed a method to automate the injection process using a stereotaxic robotic system. This study aims to demonstrate that automated injection can reliably deliver cells into the scaffold, thereby providing a more precise and reproducible method for cell seeding into grafts (Fig. 1).

## Materials and methods

### Scaffold and cell preparation

#### Decellularisation

Porcine oesophagi were harvested from 10 kg juvenile domestic pigs at the Royal Veterinary College, Hawkshead Lane, North Mymms, Hertfordshire, UK. The oesophagus was dissected *en bloc* with removal of vagal nerves. Excised oesophageal tissue was washed intra and extraluminally with Povidone iodine solution (Merck Millipore) and water.

The oesophagi were measured and cut into two halves, each approximately 5 cm in length. The mucosa was removed, and each section was decontaminated overnight at room temperature (RT) in an antibiotics/antimycotics solution: 320 mg/L gentamycin sulfate BioChemica (PanReac AppliChem), 600 mg/L clindamycin hydrochloride (Sigma-Aldrich), 500 mg/L vancomycin hydrochloride (ApexBio), 100 mg/L amphotericin B from Streptomyces sp (Sigma-Aldrich).

Porcine oesophagi were decellularised using a detergent-enzymatic protocol [8, 9]. Glass connectors were attached to the proximal and distal ends of the oesophagus with 3/0 silk purse-string sutures (Johnson & Johnson). The connectors were fixed within the glass chamber, and solutions were perfused using a peristaltic pump (i-pumps i150 Limited). For the first two days of the protocol, the oesophagus underwent a continuous wash with Milli-Q water (9 mL/min) (Days 1–2). A 4%(w/v) sodium deoxycholate (SDC, Sigma-Aldrich) solution was perfused for 4 h at RT, followed by an overnight rinse with Milli-Q water (Day 3). Twenty-five MU of DNase I (2.38%(w/v)) (EMD Millipore) in Hanks’ Balanced Salt Solution 1X (HBSS 10X, Thermo Fisher Scientific) was delivered intraluminally to the oesophagus for 3 h (3 mL/min, 37 °C) (Day 4). This process was repeated for a total of three cycles (Days 5–8), prior to a final rinse with Milli-Q water for 48 h (Days 9–10). The decellularised oesophagus was stored in Phosphate-Buffered Saline (PBS) supplemented with a single-dose antibiotic/antimycotic solution (1%(v/v)) (Gibco), and then sterilised by gamma irradiation at 1.25 kGy for 12 h, repeated for three cycles at RT. The sterile, decellularised organs were subsequently stored at 4 °C.

#### Cell culture

Human MABs and FBs were isolated from skeletal muscle donated by patients undergoing Sistrunk surgery at Great Ormond Street Hospital, London, UK, following written informed consent and with full ethical approval.

Tissue samples were washed in PBS supplemented with Penicillin/Streptomycin solution (P/S, 1%(v/v)) (Gibco), fascia was separated from muscle, and pieces were dissected into sections of approximately 1 mm³. The dissected tissue pieces were plated into cell-culture-treated wells coated with 0.15%(w/v) porcine skin gelatin (Sigma-Aldrich) and cultured in proliferation medium (PM: Megacell Dulbecco’s Modified Eagle Medium (DMEM), Sigma-Aldrich) supplemented with 5%(v/v) fetal bovine serum (FBS, Gibco), 1%(v/v) MEM non-essential amino acids 100× (Sigma-Aldrich), 2mM L-glutamine (Gibco), 1%(v/v) P/S (Gibco), 55µM 2-mercaptoethanol (Gibco), and 5ng/mL basic fibroblast growth factor (bFGF, R&D Systems). Tissue pieces were incubated at 37 °C under 5% CO₂ and 5% O₂. MABs were derived from dissected muscle samples, while FBs originated from fascia. After 3–5 days, the first outgrowths were detached using TrypLE Express (Gibco) and transferred to 0.15%(w/v) gelatin-coated flasks, with subsequent passages every 2–3 days according to cell confluence.

For scaffold injection, cell suspensions were prepared by mixing 70% human MABs and 30% human FBs, then combined in a 1:1 ratio with 15%(w/v) porcine skin gelatin (Sigma-Aldrich) to obtain a final concentration of 100,000 cells/µL.

#### Differentiation assay

For the differentiation assay, cells were plated on 0.15%(w/v) gelatin-coated wells at 16,000 cells/cm^2^ for skeletal muscle differentiation and 5,000 cells/cm^2^ for smooth muscle differentiation and cultured in PM. After 24 h, PM was replaced with differentiation medium (DM; high-glucose DMEM supplemented with L-glutamine (Gibco), 1%(v/v) P/S, and 2%(v/v) horse serum (Gibco)) for 7 days. For smooth muscle differentiation, DM was further supplemented with 5ng/mL TGF-β (Sigma-Aldrich) and refreshed daily for 6 days.

#### Immunofluorescence

Cells were fixed in 4% paraformaldehyde (PFA) at RT for 5 min, washed in PBS with Tween 20 (PBST), and permeabilised for 1 h in PBS containing 0.3% Triton X-100 (PTx.3, Thermo Fisher Scientific) at RT. Cells were then blocked in PBS with PTx.3 and 3% FBS for 1 h at RT. Primary antibodies (SM22 (ab10135, Abcam), αSMA (ab7817, Abcam), MF20 (MAB4470, R&D Systems), Calponin (C2678, Sigma-Aldrich)) were diluted in PBST with 1% (v/v) FBS at a 1:200 concentration and incubated overnight at 4 °C. After washing with PBST, cells were incubated with secondary antibodies (donkey anti-mouse and donkey anti-goat, Invitrogen) at a 1:500 concentration for 1 h at RT. Nuclei were counterstained with Hoechst 33342 (Thermo Fisher Scientific). Stained samples were imaged using a Nikon Eclipse Ti-2 microscope (Nikon).

#### Flow cytometry

Single cells were suspended in 100 µL of FACS blocking buffer (FBB: 0.2mM EDTA and 1%(v/v) FBS in PBS). Cells were incubated with 5 µL antibody solution for 30 min at 4 °C with the following fluorochrome-conjugated antibodies: CD146 FITC (Bio-Rad Laboratories), CD44 PE (Biolegend), CD90 PE-Cy7 (BD Biosciences), CD56 BUV (BD Biosciences), and CD140a BV605 (BD Biosciences). Cells were analysed in the FACSymphony A5 SE (BD Biosciences). Data were analysed by collecting a minimum of 10,000 events per sample and compared to unstained controls using the FlowJo software (BD Biosciences).

### Robotic injection

#### Robot set-up

Automated injections were performed with a stereotaxic robot developed to perform precise injections into the mouse brain (StereoDrive, Neurostar GmbH, Germany). The graft was stitched onto a custom-made 3D-printed, autoclavable rod (Form 4, Formlabs) with a diameter of 1 cm and connected to a stepper motor (Usongshine 17HS4401S, Shenzhen Youshengguangcai Electronics Co., Ltd, China), enabling automated 36° rotation of the rod. A 1mL syringe (1mL Unifix Luer Lock Syringe, Exchange Supplies) with a 30G needle (NB30G0.5, OctoInkjet Limited) was mounted on the robot’s holder.

#### Injection protocol

In this study, five injections were performed per trial. The injections were arranged in two rows, with individual injections spaced 3 mm apart and the rows separated by 3.5 mm at an angle of 36°. Samples were rotated automatically using a stepper motor.

Each injection consisted of 30 µL (3 million cells/injection), delivered at a rate of 8.8 µL/sec. The home position was first set manually on the top surface of the rod, at distance of 10 mm from the first injection point, and then all subsequent movements, including the positioning of injection sites and the control of injection volume and rate, were programmed and automated with the StereoDrive software (Neurostar GmbH) (Supplementary 1). The stepper motor, connected to the controller (Arduino Uno Rev3, Arduino, Italy), was automatically rotated by a custom Arduino programme (Supplementary 2), which controlled the rotation in response to button presses. The Arduino code was adapted to perform a 36° rotation. Since the stepper motor has a step angle of 1.8°, to achieve a total rotation of 36°, the motor must perform 20 steps.

The cell suspension described above was loaded into a syringe and maintained at 37 °C until mounted into the robot.

#### Tissue clearing

Tissue clearing followed by immunostaining was performed based on a combination of the iDISCO protocol and the BABB-based clearing method [10].

After cell injection, samples were fixed and washed in PBS. For bleaching, tissues were dehydrated through a graded methanol series (25%, 50%, 75%, and twice in 100%, 30 min each) and incubated overnight at 4 °C in methanol containing 5% hydrogen peroxide. On the following day, methanol was decreased stepwise (75%, 50%, 25%), followed by PBS and 0.2% Triton X-100 (PTx.2), before permeabilisation overnight at 4 °C in PTx.2 supplemented with 20% DMSO and 2.3% glycine. Tissues were then blocked for one day at RT in blocking solution (PTx.2, 10% DMSO and 6% FBS) and subsequently stored overnight in PBS at 4 °C.

For immunostaining, samples were incubated with anti-vimentin antibody (MAB2105, R&D Systems) (1:200) in PTwH buffer (PBS with 0.2% Tween-20 and 0.1% heparin) supplemented with 5% DMSO and 3% FBS for three days at 4 °C. After six washes in PTwH, secondary anti-rat 647 incubation (Invitrogen) (1:200) was performed overnight at 4 °C in the same buffer supplemented with 5% DMSO and 3% FBS, together with Hoechst 33,342 (2 µL/mg) nuclear stain. Tissues were then washed six times in PTwH and stored in PBS. Finally, samples were dehydrated again in a graded methanol series and subsequently cleared, first with BABB: M (methanol and BABB, 1:1) and then with BABB (benzyl alcohol: benzyl benzoate, 1:2). This clearing process rendered the tissues optically transparent. Prior to imaging, the samples were immersed in Ethyl cinnamate (Sigma-Aldrich), which acts as a refractive index matching medium to minimise light scattering and preserve tissue transparency. Imaging was then performed using the UltraMicroscope Blaze™ light sheet microscope (Miltenyi Biotec, Germany). Images were denoised and converted into an IMS format using the MACs iQ Software (Miltenyi Biotec). 3D images were rendered in Imaris software (Oxford Instruments, UK) and nuclei were automatically detected after generating a mask specific for the vimentin signal.

#### Live and dead viability assay

Cell viability after cell injection into a Petri dish using robotic and manual methods was assessed with a viability assay (Viability/Cytotoxicity Assay Kit for Animal Live & Dead Cell, Cambridge Bioscience) following the manufacturer’s instructions. Viability was assessed 3 h and 24 h post-injection. Stained samples were imaged using the Keyence bz-x810 microscope (Keyence, Japan). A viability assay also performed in decellularised tissues in which cells were injected with the robot to evaluate cell viability in situ 3 h after cell injection. Stained samples were imaged with a Nikon Eclipse Ti-2 inverted microscope (Nikon, Japan).

### Statistical analyses

All data are presented as the mean ± standard deviation. Comparisons were performed using one-way ANOVA. Statistical significance was defined as *p* < 0.05. Plots were generated using GraphPad Prism (GraphPad Software, USA).

### Figures generation

Schematics were created using BioRender (https://BioRender.com).

## Results

### Cell isolation and characterisation

**Fig. 2:**
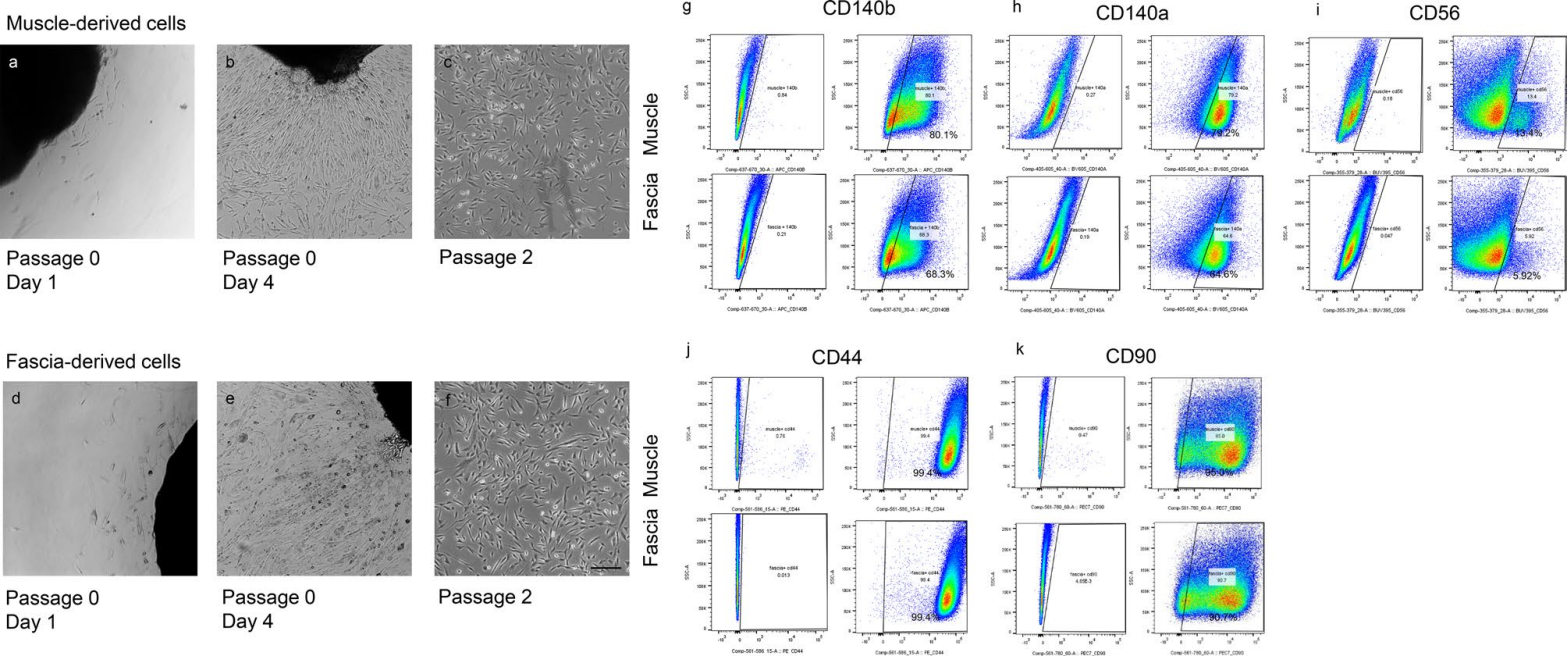
Cell isolation and characterisation. Representative brightfield images of an outgrowth derived from the muscle (**a**–**c**) and fascia (**d**–**f**). Images of the outgrowth on day 1 (**a**, **d**), day 4 (**b**, **e**) and expanded cells on passage two (**c**, **f**). Scale bar: 200 μm. Characterisation with flow cytometry of fascia-derived cells and muscle-derived cells. Unstained (left) and stained samples (right) of muscle-derived (top images) and fascia-derived (bottom images) cells stained with the markers CD140b (**g**), CD140a (**h**), CD56 (**i**), CD44 (**j**) and CD90 (**k**). The percentage of muscle and fascia-derived cells positive for each marker is indicated

**Fig. 3:**
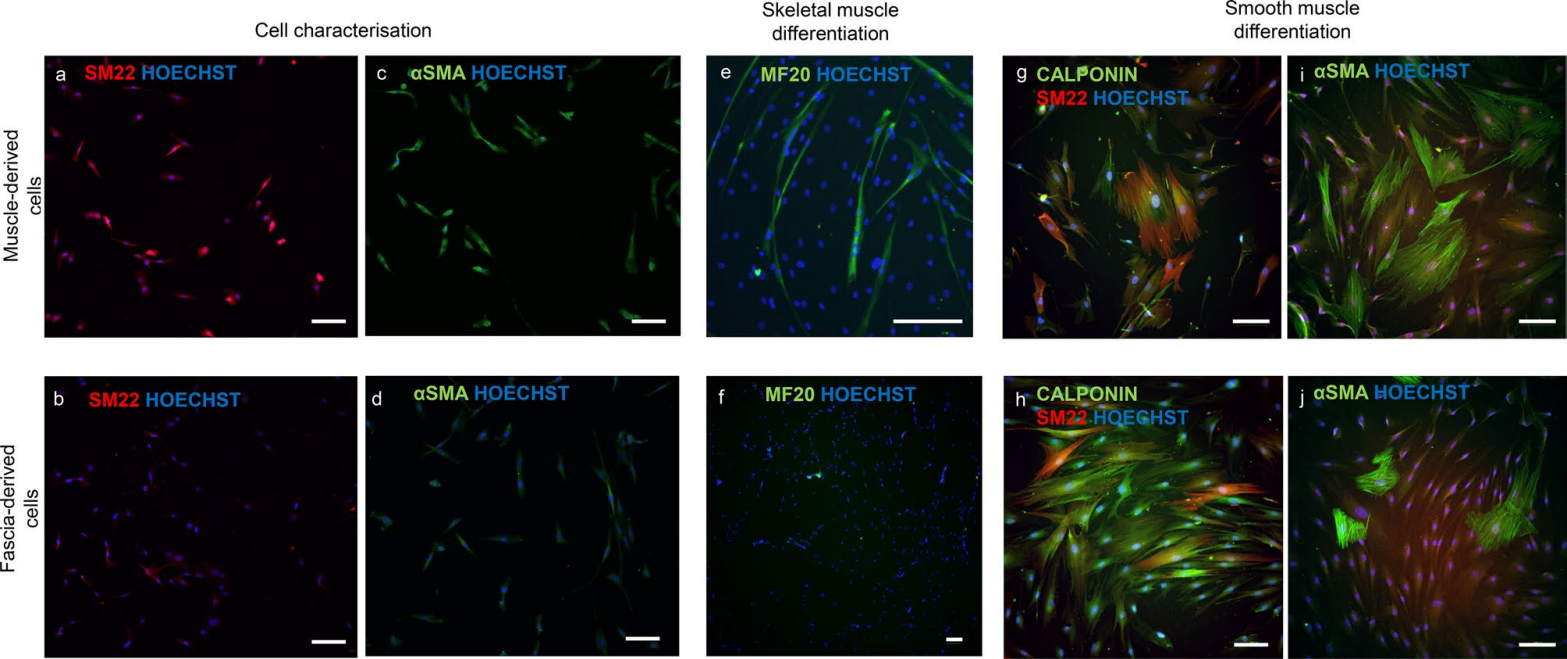
Cell characterisation and differentiation. (**a**-**d**) Immunofluorescence characterisation of muscle- and fascia-derived cells. Cells were stained for muscle progenitor markers SM22 and αSMA. (**e**, **f**) Differentiation towards skeletal muscle of muscle-derived cells after 4 days (**e**) and fascia-derived cells after 7 days (**f**) stained for MF20. (**g**-**j**) Differentiation towards smooth muscle of muscle- and fascia-derived cells after 6 days stained for calponin, SM22 and αSMA. Scale bar: 100 μm

Our graft production process relies on repopulating decellularised oesophagi with two cell types: MABs and FBs [6]. MABs are pericyte-like precursors of smooth and skeletal muscle, while FBs are known for their ability to remodel extracellular matrix (ECM) and promote MAB migration within decellularised ECM [6, 11]. We developed a protocol to derive these two cell types simultaneously from a single skeletal muscle biopsy by separating muscular and fascial tissues: muscular tissue yields muscle progenitor cells, while fascia generates bona fide FBs (Fig. 2a-f). These cells were characterised using immunofluorescence and flow cytometry. Immunofluorescent results showed that muscle-derived cells were positive for the MAB markers SM22 and αSMA, whereas fascia-derived cells were negative (Fig. 3a–d), suggesting the successful isolation and expansion of muscle progenitor cells and FBs from the biopsy after dissection. Moreover, flow cytometry results showed that muscle-derived samples had a larger percentage of cells positive for CD140b (PDGFRβ, 80.1%) and CD140a (PDGFRα, 79.2%), with a small contamination of CD56-positive cells (13.4%), supporting a MABs-like, muscle progenitor phenotype (Fig. 2g–i). Fascia-derived cells displayed lower levels of CD140b (68.3%) and CD140a (64.6%) markers with negligible levels of CD56-positive cells (5.92%); there are no specific markers for FBs. Both muscle and fascia-derived cells showed similar expression of CD44 and CD90 confirming the mesenchymal phenotype of the cells (Fig. 2j, k).

### MAB differentiation assay

Furthermore, muscle-derived cells showed differentiation towards skeletal muscle, reflected in the formation of syncytial fibres positive for MF20 (Fig. 3e), and smooth muscle cells, displayed by an enlarged cell morphology with calponin-, SM22- and αSMA-positive stress fibres (Fig. 3g, i). Partial differentiation towards smooth muscle was observed in the fascia-derived cells (Fig. 3h, j), likely due to the presence of pericyte-like cells within that population.

### Robotic microinjection into decellularised oesophageal scaffold

**Fig. 4:**
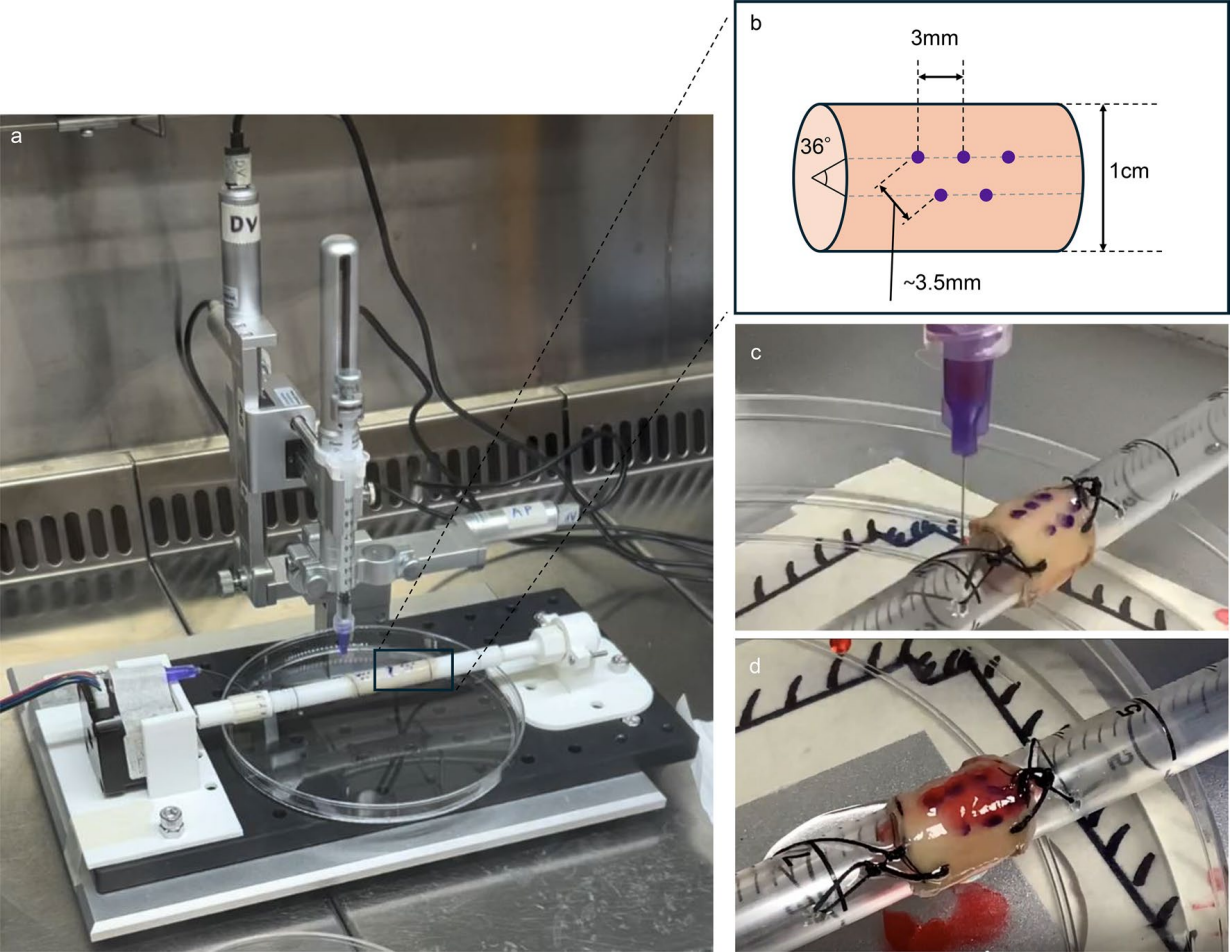
Robot setup. (**a**) Robot setup with 3D-printed autoclavable rod and stepper motor. Purple-coloured dots are labelled on the scaffold with a sterile surgical marker to help the operator visualise the injection points. (**b**) Schematics of the injection-point configuration. Optimisation of the injection protocol: injection of a 7.5% (w/v) red-coloured gelatin solution into a decellularised oesophagus (**c**) and retention of gelatin after injection (**d**), with local tissue swelling observed at the injection sites

To setup the stereotaxic robotic platform, custom-made rods were 3D-bioprinted to serve as oesophagus holders during the injections and connected to a stepper motor that allowed automated rotation of the scaffold (Fig. 4a). The injection strategy into the tissue consisted of five injections arranged in two rows, with injections spaced 3 mm apart and rows separated by 3.5 mm (Fig. 4b).

To optimise the injection sequence, a solution of 7.5%(w/v) gelatin coloured with red food dye was delivered into decellularised scaffolds (Fig. 4c, d). Prior to starting the injection, a calibration step was performed to define the distance from the holder surface, to which the tissue was mounted, taken as the reference point. Once the calibration was completed, the injection sequence was executed (Supplementary 1), resulting in the robot automatically positioning the needle over the designated sites on the scaffold. For each injection, the needle penetrated the scaffold and retracted by 10 μm before delivering the solution, creating a small cavity to facilitate the deposition of the solution. Each injection was followed by a short pause to limit backflow and fluid leakage. The robotic platform consistently positioned the needle over the predefined coordinates of the scaffold, ensuring repeatability across all five injections per trial (Fig. 4c, d). This automated positioning eliminated the need for manual adjustments.

During injection, the scaffold showed localised swelling at each targeted site, which expanded and resolved gradually as the solution dispersed within the tissue matrix (Fig. 4d). Importantly, the swelling pattern confirmed that the gelatin solution was deposited into the inner layers despite partial leaking from the surface.

Rotation of the scaffold by the stepper motor allowed the gelatin solution to be injected radially (36°), generating a uniform distribution of the solution. After each set of injections, no rupture was observed on the scaffold surface, indicating that the material could withstand repeated penetrations.

Gross examination of the scaffold following completion of the protocol demonstrated that injections resulted in multiple small, evenly spaced boluses distributed throughout the scaffold, maintaining structural integrity (Fig. 4d). Visual comparison between pre- and post-injection scaffolds revealed increased opacity at injection sites, suggesting local gelatin deposition.

### Evaluation of cell survival upon injection

**Fig. 5:**
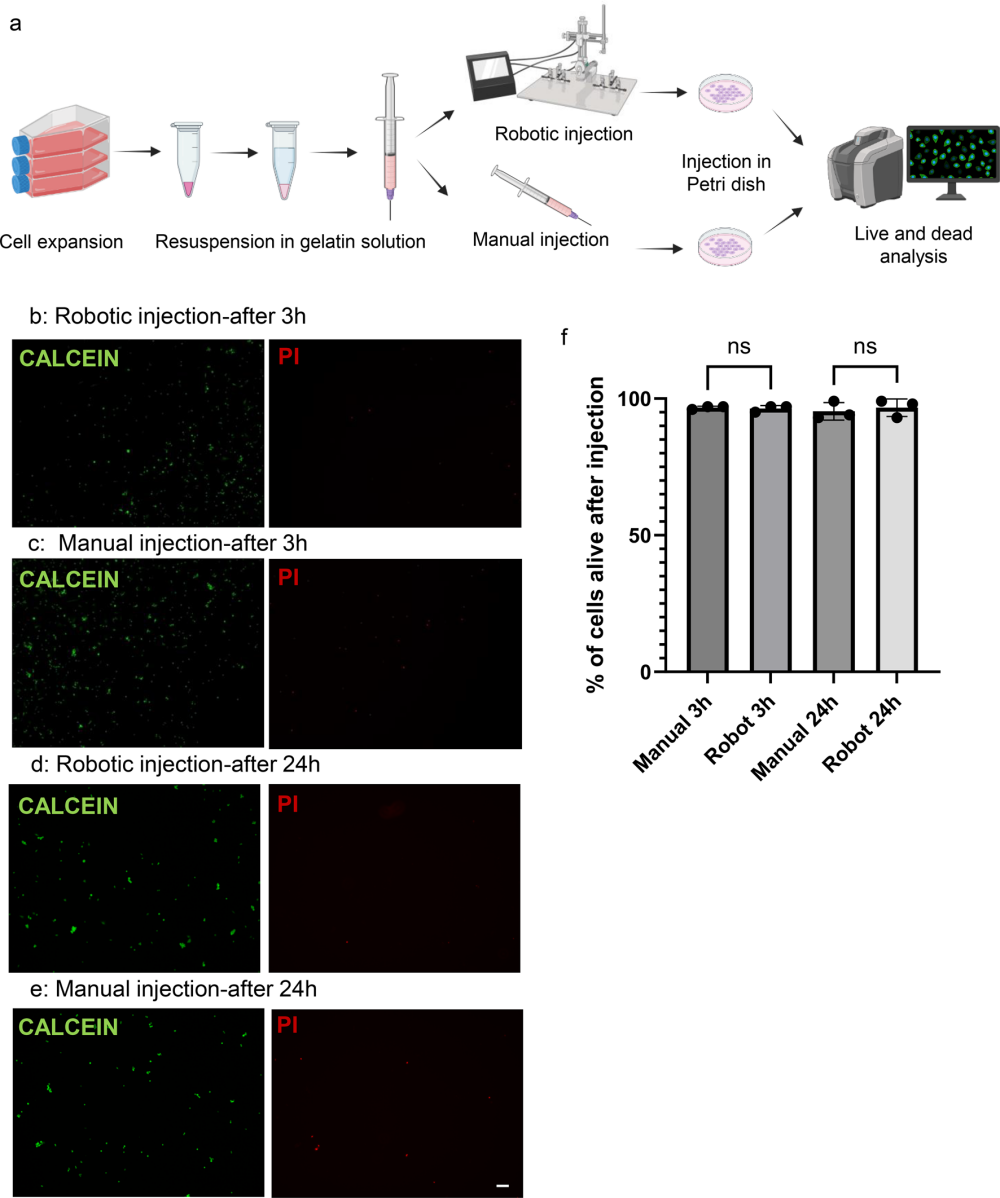
Evaluation of cell survival upon microinjection. (**a**) Schematics of the experimental setup to evaluate cell viability. Live and dead assay of cells injected with the robot and manually after 3 h (**b**, **c**) and 24 h (**d**, **e**) post-injection. (**f**) Cell viability quantification, ns = no significant differences, Scale bar: 100 μm

To analyse cell viability under the shear stress imposed by the needle, a cell viability assay was performed. Cells were injected directly into a Petri dish using both robotic and manual methods, followed by the assessment of cell viability after a 3 h and 24 h incubation post-injection (Fig. 5a). After 3 h, 97% of either robotically- or manually- injected cells remained viable (Calcein-AM positive) (Fig. 5b, c). 24 h post-injection, cell viability was 96% for robot-assisted injections (Fig. 5d) and 95% for manual injections (Fig. 5e), with no statistically significant difference observed (Fig. 5f).

**Fig. 6:**
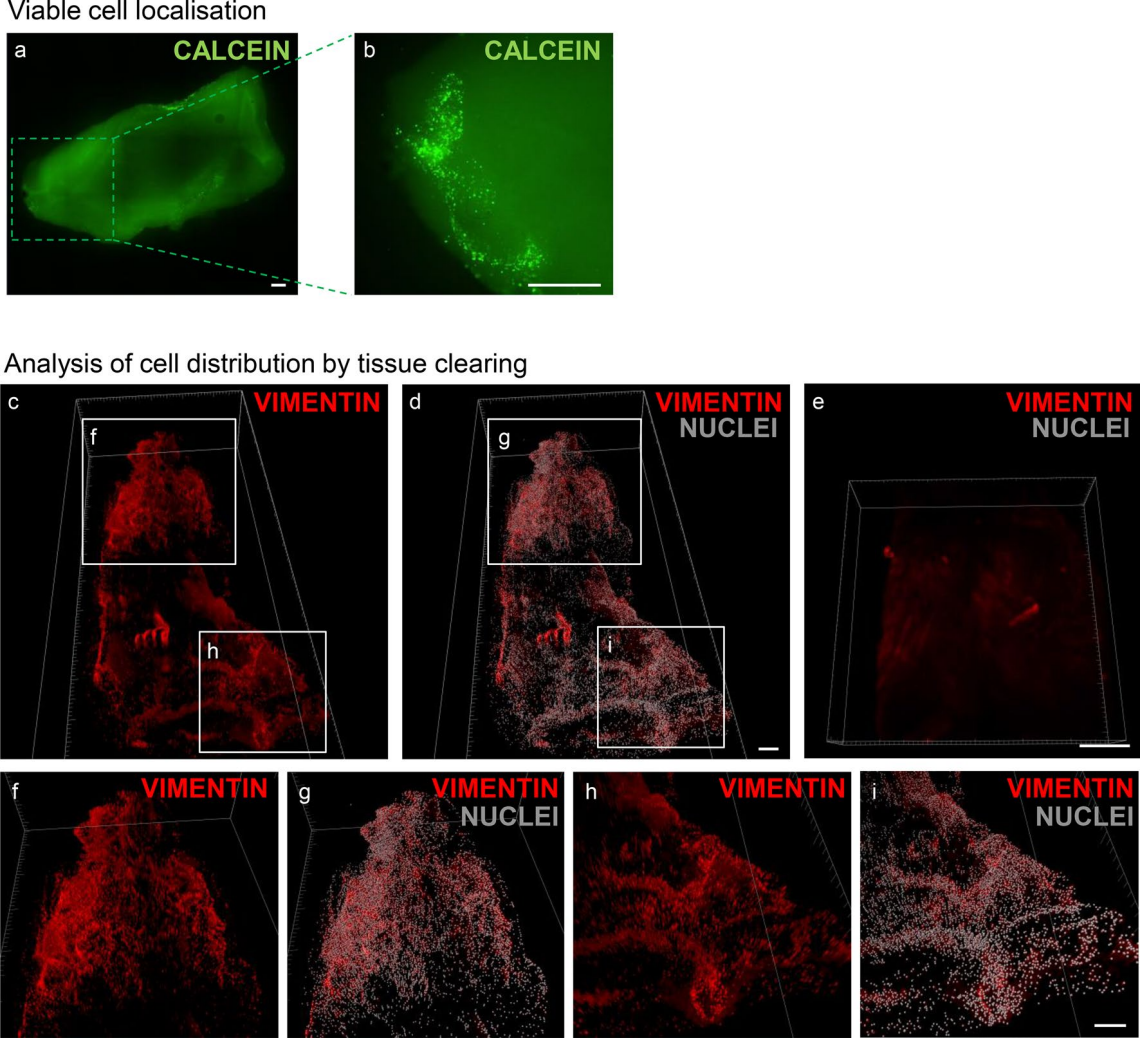
Cell localisation within the scaffold and evaluation of automatically injected cells into decellularised scaffolds. (**a**) Calcein-AM staining of cells injected within a small piece of oesophagus (1 cm x 2 cm x 1 mm) using robotic microinjection. (**b**) Zoom in of the injection point. Light sheet microscopy images of muscle- and fascia-derived cells injected automatically into a decellularised porcine scaffold using the Neurostar robot, followed by tissue clearing and staining of cells for vimentin. (**c**) Overview of the tissue and (**d**) detected nuclei using Imaris software. (**e**) Decellularised and tissue-cleared scaffold without injected cells, used as a control. (**f**-**i**) Detail of two different areas with a high density of cells and automatically detected nuclei. Scale bars: 1000 μm

To investigate whether the injected cells were retained and remained viable within the tissue after injection, the same experiment was repeated injecting the cells into a decellularised oesophagus. The majority of cells remained viable and were localised near the injection site (Fig. 6a, b).

Microscopic evaluation using light sheet microscopy following tissue clearing confirmed that injected cells were retained within the scaffold (Fig. 6c, d, f–i), whereas no cells were detected in a control sample of decellularised oesophagus that had not been injected with cells (Fig. 6e).

## Discussion

In this study, we demonstrated that a stereotaxic robotic system can reproducibly deliver cells into decellularised oesophageal scaffolds while maintaining high cell viability, thereby addressing a key limitation in the preparation of TE oesophageal grafts. Previous approaches have relied on manual seeding strategies [6–9], which are not only labour-intensive and operator-dependent but also prone to uneven cell distribution and poor reproducibility, factors that hinder both scalability and regulatory compliance.

Our findings indicate that robotic microinjection enables highly precise placement of cells with consistent depth and spacing, resulting in more uniform seeding while preserving the scaffold’s structural integrity. Importantly, cell viability following robotic injection was comparable to manual methods, demonstrating that increased technical precision does not compromise biological outcomes. This suggests that automation can be integrated into existing graft preparation workflows without altering fundamental cell-matrix interactions.

From a translational perspective, the introduction of robotics represents a step-change in the standardisation of TE graft manufacturing. Manual techniques are inherently difficult to reproduce across centres and operators, whereas robotic systems can deliver highly consistent results that are essential for moving towards regulatory approval and Good Manufacturing Practice (GMP)-compliant production. By reducing human variability, robotic injection may also lower the risk of batch-to-batch inconsistencies, facilitate multicentre trials, and accelerate the pathway from laboratory-scale proof-of-concept to clinically deployable products.

Moreover, automation creates opportunities for scale-up and high-throughput graft preparation, which will be critical if TE oesophageal grafts are to be adopted in routine clinical practice. In the long term, such systems could shorten production timelines, reduce costs, and expand patient access by making graft manufacturing more efficient and reproducible. To our knowledge, this is the first application of a stereotaxic robotic platform for oesophageal TE, and it underscores the broader potential of robotic technologies to transform regenerative medicine by bridging the gap between innovative laboratory methods and clinically viable therapies.

## Supplementary Information

Below is the link to the electronic supplementary material.

Supplementary Material 1

## Data Availability

All data supporting the findings of this study are available within the paper and its Supplementary Information.
