# Supplementary material for: Oesophageal tissue engineering: optimisation of stereotactic robotic cell injection in decellularised oesophageal scaffolds
Source: Pediatr Surg Int. 2026 Feb 20;42(1):104. doi: 10.1007/s00383-026-06309-6 (PMC12923411; doi:10.1007/s00383-026-06309-6)
Supplement: Supplementary file 1 — Supplementary Material 1 [file 383_2026_6309_MOESM1_ESM.docx]

**Supplementary 1: Sequence programmed in the StereoDrive software to automate injections.**

AP: movement of the needle in the -x axis, Lat: movement of the needle in the -y axis. Depth: movement of the needle in the -z axis. Wait1: waiting time before injection. Inj Quantity: injected volume. Inj Time: time of the injection. Wait2: waiting time after injection.

| AP | Lat | Depth | Wait1 | Inj Volume | Inj Time | Wait2 |
| --- | --- | --- | --- | --- | --- | --- |
| 0mm | 0mm | -1.5mm | 0s | 0µL | 0s | 0s |
| 10mm | 0mm | 0.01mm | 0s | 0µL | 0s | 0s |
| 10mm | 0mm | 0.00mm | 0s | 30µL | 3.4s | 5s |
| 10mm | 0mm | -1.5mm | 0s | 0µL | 0s | 0s |
| 13mm | 0mm | 0.01mm | 0s | 0µL | 0s | 0s |
| 13mm | 0mm | 0.00mm | 0s | 30µL | 3.4s | 5s |
| 13mm | 0mm | -1.5mm | 0s | 0µL | 0s | 0s |
| 16mm | 0mm | 0.01mm | 0s | 0µL | 0s | 0s |
| 16mm | 0mm | 0.00mm | 0s | 30µL | 3.4s | 5s |
| 16mm | 0mm | -10mm | 10s | 0µL | 0s | 0s |
| 14.5mm | 0mm | 0.01mm | 0s | 0µL | 0s | 0s |
| 14.5mm | 0mm | 0.0mm | 0s | 30µL | 3.4s | 5s |
| 14.5mm | 0mm | -1.5mm | 0s | 0µL | 0s | 0s |
| 11.5mm | 0mm | 0.01mm | 0s | 0µL | 0s | 0s |
| 11.5mm | 0mm | 0.0mm | 0s | 30µL | 3.4s | 5s |
| 11.5mm | 0mm | -1.5mm | 0s | 0µL | 0s | 0s |
| 0mm | 0mm | -20mm | 10s | 0µL | 0s | 0s |

**Supplementary 2: Custom Arduino program to control the rotation of the stepper motor.**

const int stepPin = 2 ;

const int dirPin = 5;

const int buttonPin = 4;

int x;

void setup() {

pinMode(stepPin, OUTPUT);

pinMode(dirPin, OUTPUT);

pinMode(buttonPin, INPUT_PULLUP);

x=0;

}

void loop() {

digitalWrite(dirPin, HIGH);

int buttonState = digitalRead(buttonPin);

if (buttonState == LOW){

for (int x = 0; x < 20; x++){

digitalWrite(stepPin, HIGH);

delayMicroseconds(500);

digitalWrite(stepPin, LOW);

delayMicroseconds(500);

}

delay(1000);

}

else {

digitalWrite(stepPin, LOW);

}

}
